# 17*β* Estradiol Modulates Perfusion Pressure and Expression of 5-LOX and CYP450 4A in the Isolated Kidney of Metabolic Syndrome Female Rats

**DOI:** 10.1155/2015/149408

**Published:** 2015-09-27

**Authors:** A. M. Zúñiga-Muñoz, V. Guarner Lans, E. Soria-Castro, E. Diaz-Diaz, R. Torrico-Lavayen, E. Tena-Betancourt, I. Pérez-Torres

**Affiliations:** ^1^Department of Pathology, Instituto Nacional de Cardiología “Ignacio Chávez”, Juan Badiano No. 1, Sección XVI, 14080 Tlalpan, DF, Mexico; ^2^Department of Physiology, Instituto Nacional de Cardiología “Ignacio Chávez”, Juan Badiano No. 1, Sección XVI, 14080 Tlalpan, DF, Mexico; ^3^Department of Reproduction Biology, Instituto Nacional de Ciencias Médicas y Nutrición “Salvador Zubirán”, Vasco de Quiroga 15, Sección XVI, 14000 Tlalpan, DF, Mexico; ^4^Animal Facility Services and Experimental Surgery, Facultad de Medicina Universidad La Salle, Avenue De las Fuentes 17, 14000 Tlalpan, DF, Mexico

## Abstract

Prevalence of metabolic syndrome and progression of nephropathy depend on sex. We examined a protective effect of estradiol against nephropathy in metabolic syndrome through the modulation of the arachidonic acid metabolism by activating the 5-lipoxygenase and cytochrome p450 4A pathways. 28 female Wistar rats were divided into four groups of seven animals each: control, intact metabolic syndrome, ovariectomized metabolic syndrome, and metabolic syndrome ovariectomized plus estradiol. Blood pressure, body weight, body fat, triglycerides, insulin, HOMA-index, albuminuria, and TNF-*α* were increased in ovariectomized metabolic syndrome rats (*p* < 0.001). The perfusion pressure in isolated kidneys of ovariectomized metabolic syndrome rats in presence of 4 *μ*g of arachidonic acid was increased. The inhibitors of the arachidonic acid metabolism Baicalein, Miconazole, and Indomethacin in these rats decreased the perfusion pressure by 57.62%, 99.83%, and 108.5%, respectively and they decreased creatinine clearance and the arachidonic acid percentage. Phospholipase A_2_ expression in the kidney of ovariectomized metabolic syndrome rats was not modified. 5-lipoxygenase was increased in metabolic syndrome ovariectomized rats while cytochrome p450 4A was decreased. In conclusion, the loss of estradiol increases renal damage while the treatment with estradiol benefits renal function by modulating arachidonic acid metabolism through the 5-lipoxygenase and cytochrome p450 4A pathways.

## 1. Introduction

Metabolic syndrome (MS) is a cluster of various metabolic pathologies including hypertension, dyslipidemia, hyperinsulinemia, insulin resistance (IR), and central obesity [1]. MS is accompanied by a prothrombotic and a proinflammatory state [2] that increases the risk of the renal function abnormalities [3]. The prevalence of MS depends on sex and there are sex differences in the development and progression of nephropathy [3]. In nephropathy models in humans and animals, males with MS exhibit a faster decline in renal function than females. The incidence of MS is lower in premenopausal women and it increases after menopause, when estrogen levels dramatically decrease accelerating the progression of atherosclerosis and kidney diseases [4]. These differences could be explained by the presence of estradiol (E_2_). This hormone can decrease vascular resistance and inhibit circulating renin and angiotensin converting enzyme thereby decreasing circulating angiotensin levels [5]. It suppresses the synthesis of proteins that are increased by nephropathies such as collagen and fibronectin [5, 6]. Supplementation with E_2_ also prevents albuminuria, glomerulosclerosis, and tubulointerstitial fibrosis associated with diabetic nephropathy [7].

One of the mechanisms through which E_2_ exerts renoprotection is by regulating the expression of extracellular matrix proteins [8]. It also decreases the accumulation of LDL in coronary arteries and activates muscarinic receptors, thus stimulating endothelial cells to synthesize nitric oxide (NO) and, consequently, increasing the circulating levels of nitrates and nitrites [9]. E_2_ reduces the influx of extracellular Ca^2+^ into vascular smooth muscle cells [10]. It decreases the affinity of receptors to vasoconstrictor substances and has a relaxing effect on vessels, decreasing levels of endothelin and norepinephrine [11]. It also has antioxidant properties against reactive oxygen species [12].

Arachidonic acid (AA) metabolites, also known as eicosanoids, represent a large class of lipid mediators that exert diverse and complex functions such as maintenance of body fluid homeostasis, normal kidney function, and blood pressure [13]. These lipid mediators also play an important role in the pathophysiology of the inflammatory process, hypertension, and kidney diseases [13]. The synthesis of eicosanoids is mediated by three enzymatic pathways: cyclooxygenase isoforms (COX-1 and COX-2), lipoxygenase (5-, 12-, and 15-LOX), and cytochrome P450 (CYP450 4A, 2C2, 2C6, and 2J2). Eicosanoids include prostanoids derived from the COX pathways, leukotrienes synthetized by the LOX pathways, and hydroxyeicosatetraenoic acids (HETEs) and epoxyeicosatrienoic acids (EETs) derived from the CYP450 4A and 2C2, 2C6, and 2J2 pathways, respectively [4].

Previous research in our laboratory showed that the increase in perfusion pressure (PP) in isolated kidney of MSovx rats in presence of 4 *μ*g AA was related to a decrease in COX-1 and overexpression of COX-2 and that replacement with E_2_ reverted it [4]. Other studies have shown that E_2_ blocks the increased expression of COX-2 in the vessels and that it inhibits the increased production of prostaglandin E_2_ in response to inflammatory stimuli [14]. However, there are few studies on the 5-LOX and CYP450 4A pathways in renal disease. Furthermore, the effect of the lack of E_2_ upon the expression of these enzymes has not been studied. The present work in the MS female rat model provides information on these issues. Our MS rat model is a variant of that described by Reaven and Ho [15]. In this model the animals develop hypertension, hypertriglyceridemia, central obesity, IR, alterations in vascular reactivity, and renal damage [16] after consumption of 30% sucrose in drinking water for 24 weeks [17]. Our aim was to investigate whether the presence of estrogen modulates the perfusion pressure (PP) in isolated perfused kidneys and if the expression of 5-LOX and CYP450 4A are related to the modulation of PP.

## 2. Materials and Methods

### 2.1. Animals

Experiments in animals were approved by the Laboratory Animal Care Committee of our institution and were conducted in compliance with the Guide for the Care and Use of Laboratory Animals. 28 female Wistar rats reared in the National Institute of Cardiology (Mexico) vivarium were housed with a 12:12 light-dark cycle used. Water and rodent commercial food (23% of crude protein, 4.5% of crude fat, 8% of ashes, and 2.5% of added minerals) were given to the animals ad libitum.

### 2.2. Experimental Protocol

Rats were randomly divided into four groups of seven animals each.

*Group 1*. Control (C) rats: rats were given tap water for drinking and standard commercial food. 

*Group 2*. Intact metabolic syndrome (MS) rats: rats were given drinking water containing 30% of sucrose for 24 weeks beginning when animals reached approximately 6 to 7 weeks of age (150 ± 5 g). 

*Group 3*. Ovariectomized metabolic syndrome rats (MSovx): rats weighing 100 g were ovariectomized before the onset of reproductive cycles. They were then given sucrose as in the previous group. 

*Group 4*. Ovariectomized MS plus E_2_ rats (MSovx + E_2_): rats were ovariectomized and began the sucrose treatment as in the previous group. The E_2_ treatment was begun 3 days after the ovariectomy. Rats received ip injections of Valerate (Primogyn, Schering, Mexico, 1 mg/Kg body weight) every 3 days during the 24 weeks of the sucrose treatment, therefore mimicking the reproductive cycles of intact rats. The frequency of the E_2_ treatment was chosen after performing an E_2_ concentration decay assay (data not shown). Estrogen level was decreased according to the phases of the reproductive cycle in the rat in which there is a peak of E_2_ approximately every 3 to 5 days during the phases of estrous [18]. This group constitutes an ideal control group since it underwent the surgical procedure and its consequences except for the absence of E_2_ which was given as a pulsatile hormone replacement treatment. Although the amount of E_2_ injected was high, the serum levels were similar to those in the control and MS group (Table 1).

During the last 5 days of the sucrose treatment period, the animals were placed in metabolic cages (Nalgene, San Diego, CA) for 5 days, with free access to food and water or sucrose solution, and urine was collected. The urine was filtered and collected on ice for 24 hours. After the 24 weeks of sucrose treatment all of the animals were weighed and the blood pressure was measured. The animals were sacrificed and serum and the kidneys were obtained.

### 2.3. Ovariectomy

The animals were fasted overnight and then anesthetized by intraperitoneal injection of sodium pentobarbital (63 mg/kg, Pfizer, Mexico City, Mexico).

The abdominal and pelvic area of the back was depilated, cleaned with soap, and disinfected with ethanol. A longitudinal incision of 1.5 cm was made, the skin was separated from the muscle, and a second incision of 0.5 cm was made in the muscle on both sides of the first, to exteriorize the ovaries. The Fallopian tubes were ligated and cut below the ligature. After the extirpation, the incision was sutured [4].

### 2.4. Systolic Blood Pressure

Systolic blood pressure (SBP) was measured by the tail-cuff method [6].

### 2.5. Albuminuria and Urine Creatinine

Albuminuria was measured using bromocresol green reagent. This technique is specific for the quantification of albumin in urine [19]. Urine creatinine was measured by the Jaffe method [20].

### 2.6. Serum Sample

The abdominal aorta was exposed by midline laparotomy and cannulated to obtain 4 mL of blood, taking care to avoid haemolysis. The blood was centrifuged for 20 minutes at 600 g and at 4°C. The serum was separated and stored at −30°C.

### 2.7. Biochemistry and Measurement of Serum E_2_

The measurements of cholesterol, triglycerides (TG), and glucose were carried out with enzymatic kits (Pointe Scientific Inc. Canton, Michigan, USA). Serum insulin was evaluated using specific kit (Linco Research, Inc. Missouri, USA). The HOMA-IR index for IR was calculated (HOMA-IR = [Insulin *μ*U/mL] *∗* [Glucose mM]/22.5) [21]. Serum creatinine was measured by Jaffe method [20]. Serum E_2_ was measured using the Diagnostic Products Corporation kit (Los Angeles, CA).

### 2.8. Isolated Perfused Kidney

The right kidney was exposed by midline laparotomy, and the mesenteric and right renal arteries were cleared of surrounding tissue. The right renal artery was cannulated through the mesenteric artery to avoid interruption of blood flow; and the kidney was removed, suspended, and perfused at constant flow by means of a peristaltic pump (MasterFlex Easy-load II, number 77200-50; Cole-Parmer Instrument Co, Vemon Hills, IL) with Krebs solution at 37°C and oxygenated with 95% O_2_/5% CO_2_. The solution had the following composition (mM/L): 118 NaCl, 1.2 NaH_2_PO_4_, 25 NaHCO_3_, 4.7 KCl, 1.2 CaCl_2_, 4.2 MgSO_4_, and 5.5 glucose (pH 7.4). Flow was adjusted to a basal perfusion pressure (PP) of 75 to 90 mmHg. Mean flow rate of the perfusing solution was 8 to 9 mL/min. PP was measured with a transducer (Grass Telefactor, Grass Technologies, Astro Med, West Warwick, RI), captured, and recorded by means of a Grass model polygraph 79D and an online program (Grass PolyView Data Acquisition and analysis version 2.0). Changes in the PP produced by AA were calculated by taking the mean of the pulsatile trace before the administration of AA and the mean of the trace at the maximal PP value after injection of AA. Data are expressed as changes (Δ) of PP in mmHg [4]. After at least 15 minutes of perfusion and once a stable PP had been obtained, vasoconstrictor responses to AA 4 *μ*g/mL·min were determined in the absence and presence of 10 *μ*mo/L Baicalein (5-LOX pathway inhibitor), 5 *μ*mo/L Miconazole (pathway inhibitor of CYP450 4A), or 10 *μ*mo/L Indomethacin (COX isoforms pathway inhibitor). The doses of AA (4 *μ*g/mL·min) and Indomethacin (10 *μ*mo/L) were selected as the most effective from published data [4]. After each perfusion bolus of AA, in the absence and presence of inhibitors, the kidneys were washed for a period of 20 min with Krebs solution, to allow the return to their basal PP (75–90 mmHg), and no sign of tachyphylaxis was present. AA sodium salt was dissolved in double distilled water; Indomethacin was dissolved in Na_2_CO_3_ 1.5 mmol/L; Baicalein and Miconazole were dissolved in CH_3_-OH.

### 2.9. Kidney Homogenate

The left kidney was dissected and washed with saline solution and immediately perfused with sucrose buffer (25 mM sucrose, 10 mM Tris, 1 mM EDTA, pH 7.35). The capsule was removed and the kidney was homogenized in the same sucrose buffer with protease inhibitors (1 mM PMSF, 2 *μ*M pepstatin, 2 *μ*M leupeptin, and 0.1% aprotinine). The homogenate was kept on ice. The kidney homogenate was centrifuged at 900 g for 10 min at 4°C. The supernatant was separated and stored at −30°C until required. Total proteins were determined by Lowry method [22].

### 2.10. Histopathological Analysis

For histology, small pieces of kidney cortexes before homogenization were processed according to conventional histological procedures for electron microscopy. The sample was fixed in 2% paraformaldehyde and 2.5% glutaraldehyde for 1 hour and then stored in 0.1 mM cacodylate buffer (pH 7.4). They were then postfixed in 1% osmium tetroxide in 0.1 mM cacodylate buffer (w/v). The samples were dehydrated in a graded series of ethanol and embedded in EPON 812 (Electron Microscopy Sciences). Ultrathin sections (approximately 60 nm in thickness) were cut using a Leica Ultracut microtome and mounted on copper grids. Sections were contrasted with uranyl acetate and lead citrate. Histological sections were analyzed using an electron microscope JEOL-1011 (JEOL Ltd., Tokyo, Japan) at 60 kV. Random pictures of 2-3 glomeruli were taken from 7 rats per group, using 20000x magnifications.

### 2.11. Immunoblotting

100 *μ*g of kidney homogenate was mixed with sample buffer (Tris-HCl, 1% sodium dodecyl sulfate, 50% glycerol, and 0.1% bromophenol blue, pH 6.5) and boiled for 2 minutes. Proteins were separated on a 10% SDS–PAGE and transferred to Hybond-C extra nitrocellulose membrane (Millipore). The blots were blocked for 3 hours with Tris-buffer solution (TBS) containing 5% nonfat dry milk and 0.5% Tween 20. A rabbit Phospholipase A_2_ (PLA_2_), 5-LOX, and CYP450 4A polyclonal antibody (Santa Cruz Biotechnilogy, Santa Cruz, Ca) were applied individually to each gel, at a dilution of 1 : 1000, for an entire night. The blots were washed in TBS and incubated with secondary antibody biotinylated-goat anti-rabbit immunoglobulin (ZYMED Laboratories, San Diego, CA) at a dilution of 1 : 5000.

After incubation with the secondary antibody, the membranes were washed with TBS and the band detection was carried out using 3′3′-diaminobenzidine. Membranes were stripped in a TBS containing 1% SDS and 100 mmol/L *β*-mercaptoethanol (pH 2), followed by incubation with a 1/2000 *α*-actin monoclonal mouse antibody. Band intensity was measured densitometrically with ID Kodak Image Analysis Software, Windows Version 3.5.

### 2.12. Arachidonic Acid of the Phospholipids from Kidney Homogenate

To determine the AA of the phospholipids, 100 *μ*L of the homogenized kidney solution was used in the presence of 100 *μ*g nonadecanoic acid as internal standard and 200 *μ*L of acetone. The mixture was shaken vigorously for 30 seconds in a vortex and centrifuged at 1145 g, at room temperature for 4 minutes. The supernatant was removed and the pellet was suspended with 2 mL of CH-Cl_3_/CH_3_-OH (2 : 1, vol/vol) with 0.002% BHT, as described by Folch et al. [23]. AA were transesterified to their AA methyl esters by heating them at 90°C for 2 h with methanol, 2% concentrated H_2_SO_4_, and 0.002% BHT. AA methyl esters were separated and identified by gas chromatography-FID in a Carlo Erba Fratovap 2300 chromatograph equipped with a capillary column packed with the SP-2330 phase (30 m long and 0.25 mm, 0.2 mm film thickness) and fitted with a flame ionization detector at 210°C, with helium as the carrier gas at a flow rate of 1.2 mL/min.

### 2.13. Cytokines

IL-1*α*/IL-1F1, IL-4, IL-10, and TNF-*α* in kidney homogenates were determined by ELISA kits obtained from Elabscience Biotechnology Co., Ltd.

### 2.14. Statistical Analyses

Statistical analysis and graphics were performed with a SigmaPlot 11 program. The data are presented as the mean ± SE. Statistical significance was determined by two-way ANOVA test, followed by the post hoc Tukey test. Differences were considered statistically significant at *p* < 0.05.

## 3. Results

### 3.1. Effects of Ovariectomy on SBP, Body Variables, and Serum Biochemical Measurements

Table 1 shows that body weight, intra-abdominal fat, SBP, triglycerides, insulin, and HOMA index were significantly higher in the MS group than in the C group (*p* < 0.001) and that they were further increased in the MSovx group (*p* < 0.001). The levels of these variables were similar to those in the MS group when ovariectomy was followed by E_2_ treatment. None of the groups showed a significant difference in the levels of cholesterol, glucose in serum, and kidney weight. Albuminuria was not altered in C, MS, and MSovx + E_2_ groups; but it showed a significant increase in the MSovx group (*p* < 0.05).

No significant difference in E_2_ concentration between C, MS, and MSovx + E_2_ groups was found. However, the E_2_ concentration in MSovx group was lower than that of the MS and MSovx + E_2_ groups (*p* < 0.001) (Table 1).

The results of the urinary and serum creatinine determinations in each group were used to calculate the creatinine clearance thus evaluating the renal function. Results showed that the MSovx group had a significant decrease in its creatinine clearance when compared to that in the MS and MSovx + E_2_ groups (*p* < 0.01). The difference in creatinine clearance was not statistically significant when comparing the C group against the MS groups (Table 1).

### 3.2. Arachidonic Acid of the Phospholipids Percentage from Kidney Homogenate

**Figure 1:**
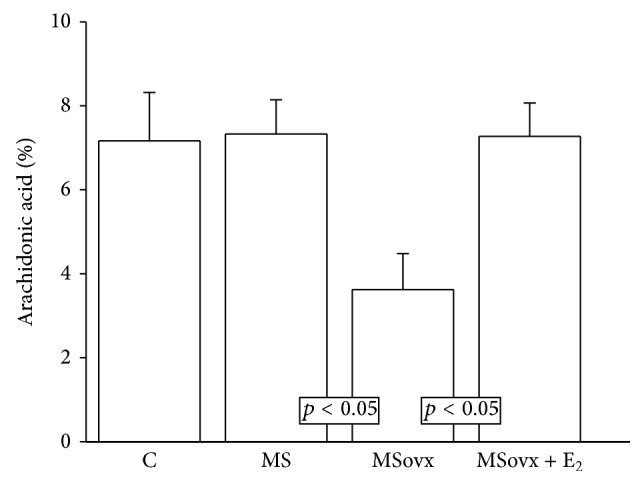
Effect of the estrogen removal and estradiol replacement on the percentage of arachidonic acid in the phospholipids from kidney homogenates. C: control, MS: metabolic syndrome, MSovx: metabolic syndrome ovariectomized, and MSovx + E_2_: metabolic syndrome ovariectomized plus estradiol. Data are means ± SE; *n* = 7 in each group. Comparison between MS and MSovx versus MSovx + E_2_.

Figure 1 shows that the difference in the percentage of AA in the phospholipids from the kidney homogenates of the C, MS, and MSovx + E_2_ groups was not significant; however, it was significantly decreased in the kidney homogenate from MSovx group (3.62 ± 0.86 versus 7.33 ± 0.81, resp., *p* < 0.05).

### 3.3. Arachidonic Acid Effect on Δ Perfusion Pressure in Isolated Kidney

**Figure 2:**
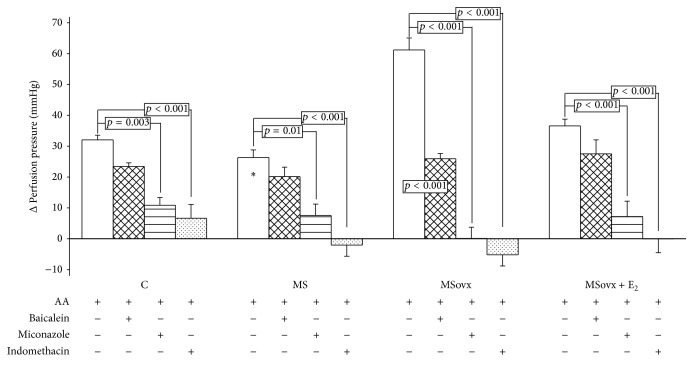
Arachidonic acid effect on Δ perfusion pressure in isolated kidney from experimental groups. Kidneys were perfused with 4 *μ*g of AA in the absence or the presence of inhibitors. AA 4 *μ*g, AA 4 *μ*g plus 10 *μ*mo/L Baicalein, AA 4 *μ*g plus 5 *μ*mol/L Miconazole, and 1 AA 4 *μ*g plus 10 *μ*mo/L Indomethacin. C: control, MS: metabolic syndrome, MSovx: metabolic syndrome ovariectomized, and MSovx + E_2_: metabolic syndrome ovariectomized plus estradiol. Data are means ± SE; *n* = 7 in each group. ^*∗*^MS versus MSovx *p* < 0.001.

The ΔPP was measured in perfused isolated kidneys from C, MS, MSovx, and MSovx + E_2_, with 4 *μ*g of AA.  Figure 2 shows that there was no difference in ΔPP between C, MS, and MSovx + E_2_ groups (32.04 ± 1.50, 26.30 ± 2.50, and 36.58 ± 2.18 mmHg, resp.). However, when comparing the ΔPP of kidney from MSovx (61.17 ± 3.88 mmHg) with that of the MS and MSovx + E_2_ groups, it was significantly higher (*p* < 0.001).

The perfusion of 4 *μ*g of AA plus 10 *μ*mo/L Baicalein decreased the ΔPP in kidney of MSovx group in a 57.62% (*p* < 0.001), but it did not modify the ΔPP in the kidney from C, MS, and MSovx + E_2_ (Figure 2).

Figure 2 shows the ΔPP when 4 *μ*g of AA and Miconazole 5 *μ*mo/L were perfused in the kidney from C, MS, MSovx, and MSovx + E_2_ groups. The ΔPP decreased by 66.09% (*p* = 0.003), 71.28% (*p* = 0.01), 99.83% (*p* < 0.001), and 80.29% (*p* < 0.001), respectively, in comparison to the ΔPP without Miconazole. There was no significant difference in the ΔPP with Miconazole between the experimental groups.

When 4 *μ*g of AA and 10 *μ*mo/L of Indomethacin were perfused in the kidney from C, MS, Movx and, Movx + E_2_ groups, a decrease in the ΔPP of 79.18% (*p* < 0.001), 107.78% (*p* < 0.001), 108.5% (*p* < 0.001), and 100.12% (*p* < 0.001), respectively, was observed in comparison with the ΔPP without Indomethacin. There was no significant difference in the ΔPP with Indomethacin between experimental groups.

### 3.4. 5-LOX, CYP450 4A, and PLA_2_ Expression

**Figure 3:**
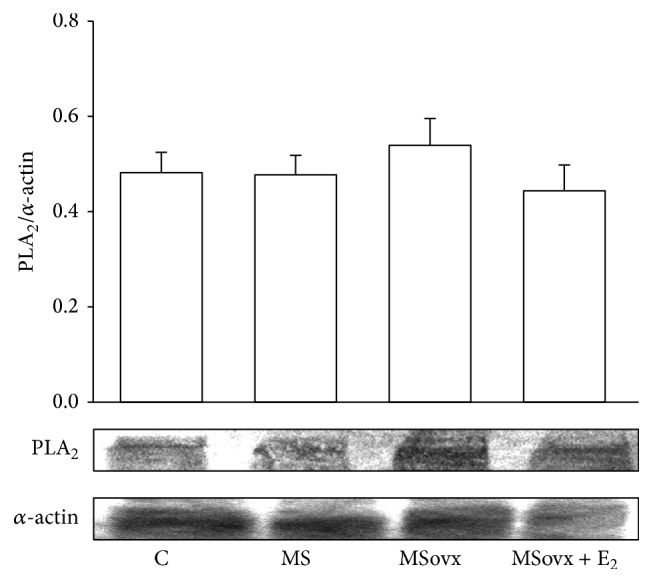
Representative densitometric analysis of Western blots for PLA_2_ protein expression in kidney homogenate in experimental groups. Data are means ± SE; *n* = 7 in each group.

**Figure 4:**
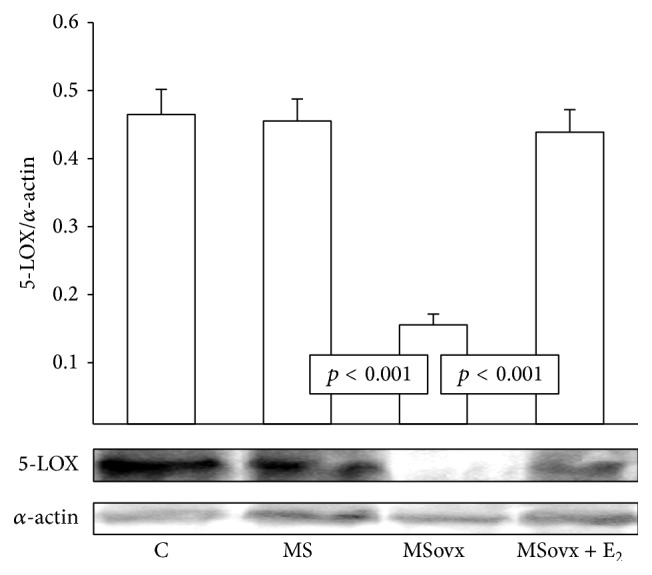
Densitometric analysis of Western blots for 5-LOX protein expression in kidney homogenate in experimental groups. Data are means ± SE; *n* = 7 in each group.

**Figure 5:**
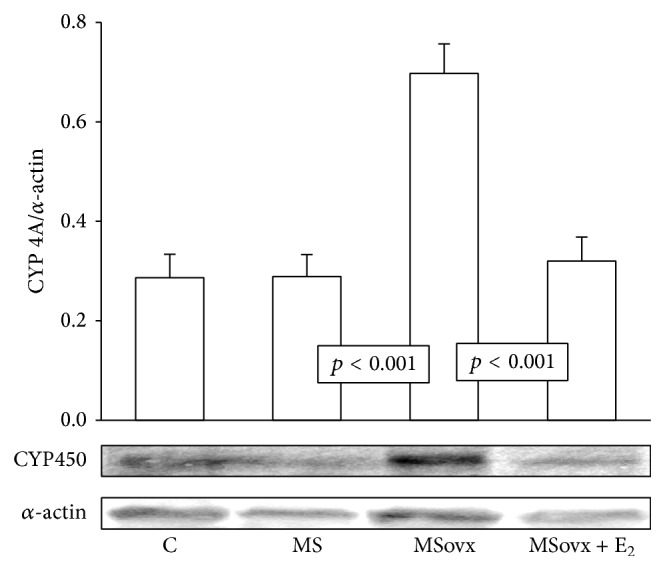
Representative densitometric analysis of Western blots for CYP450 4A protein expression in kidney homogenate in the experimental groups. C: control, MS: metabolic syndrome, MSovx: metabolic syndrome ovariectomized, and MSovx + E_2_: metabolic syndrome ovariectomized plus estradiol. Data are means ± SE; *n* = 7 in each group.

None of the groups showed a significant difference in the PLA_2_ protein expression (Figure 3). 5-LOX protein expression in kidney homogenate from the MSovx group was significantly decreased in comparison to MS and MSovx + E_2_ groups (*p* < 0.001). No significant differences in 5-LOX protein expression in the other groups were observed (Figure 4).  Figure 5 shows a significant increase in CYP450 4A expression in the kidney homogenate from the MSovx group in comparison to that in the MS and MSovx + E_2_ groups (*p* < 0.001). There was no significant difference in the CYP450 4A protein expression among the other groups.

### 3.5. Cytokines

IL-10 in kidney homogenate shows a significant decrease in the MSovx group when compared to that in the MS and MSovx + E_2_ groups (*p* < 0.01 and *p* < 0.001, resp.). TNF-*α* in the kidney homogenates showed a significant increase in the MS group in comparison to that of the C group (*p* < 0.001). Nevertheless, TNF-*α* shows a significant increase in the MSovx and MSovx + E_2_ groups when compared to that in the MS group (*p* < 0.001). IL-4 showed a tendency to decrease in MSovx but the difference was not statistically significant in comparison to that of the MS group. The IL-1*α*/IL-1F1 ratio did not show statistically significant differences in any of the groups studied (Table 2).

### 3.6. Electron Microscopy Histology

**Figure 6:**
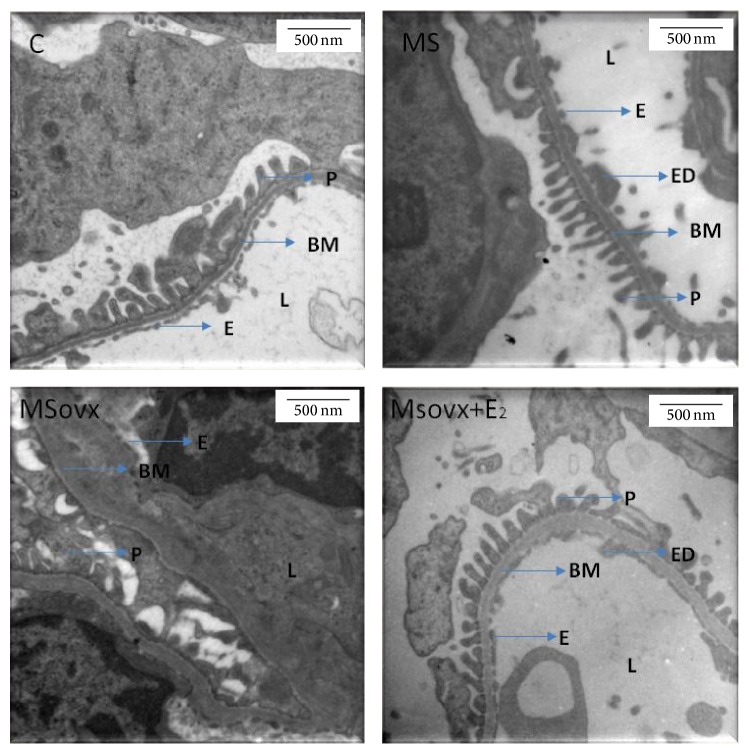
Representative micrographs of electron microscopy of renal capillary loops (20000x). C rats, no abnormalities are found. The images from MS and MSovx rats show abnormalities in basal membrane with endothelial edema and filiform podocyte in some zones. The images from the MSovx + E_2_ rats show abnormalities in basal membrane with remodeling and thickness variability; the podocyte processes have almost disappeared and the fenestrated endothelium is lost or obliterated in many areas. C: control, MS: metabolic syndrome, MSovx: metabolic syndrome ovariectomized, MSovx + E_2_: metabolic syndrome ovariectomized plus estradiol, BM: basal membrane, E: endothelium, ED: edema, L: lumen, and P: podocyte.

The micrographs of the kidneys from the MS rats showed abnormalities in the basal membrane with endothelial edema and filiform podocytes in some zones when compared to those from the C rats. The micrographs of MSovx rats showed abnormalities in basal membrane with remodeling and thickness variability. The podocyte processes almost disappeared and the fenestrated endothelium was lost and obliterated in many areas in comparison to micrographs from MS rats. The micrographs of MSoxv + E_2_ rats show similar characteristics to those from the MS rats (Figure 6).

## 4. Discussion

### 4.1. Body Weight, Intra-Abdominal Fat, and Glucose

We studied the modulation by E_2_ of the AA metabolism through the 5-LOX and CYP450 4A pathways in the kidney in the MS female rats. The increase in body weight and intra-abdominal fat observed in female MSovx rats may be due to loss E_2_ levels. In ovariectomized and aromatase knockout mice, body weight is gained and obesity is developed, suggesting that E_2_ plays an important role in the regulation of energy balance. Body weight and intra-abdominal fat elevations were attenuated when replacement with E_2_ was given [24]. Ovariectomy and E_2_ therapy also modified energy balance and body fat content in Syrian hamsters without modifying their food intake [25, 26]. Another study showed that after 10 weeks of consuming a high fat diet, male mice had significantly higher body weight than intact female mice. Male mice showed similar changes in body weight to those in Ovx females. When E_2_ was supplemented, the alterations in body weight were minimal [27]. Furthermore, it has been reported that E_2_ regulates body weight and energy metabolism by acting on the brain in a way similar to that of leptin [28]. We did not find significant differences in the glucose concentrations in any of our experimental groups since our model has no alterations in glucose metabolism [4, 6, 29]. When glucose uptake is stimulated, sucrose fed rats are more insulin resistant and therefore the serum glucose concentration does not change. Similar results have been described by Reaven and Ho [15].

### 4.2. Hypertriglyceridemia and Hyperinsulinemia

The hypertriglyceridemia observed in MSovx group could be associated with obesity and E_2_ deficiency. It has been proposed that the hepatic metabolism of fructose may be different when E_2_ is absent or that clearance of TG may be accelerated when compared to that in intact female rats [12]. Investigations have shown a significant increase in TG in MS female rats obtained by administering a high-fat diet (60%) over a period of 14 weeks. In addition, the loss of E_2_ can increase the activity of the hormone-sensitive lipase. This enzyme participates in the release of fatty acids, which might in turn be reflected in the increase of TG [30]. Hypertriglyceridemia has been associated with IR and hyperinsulinemia. The HOMA index increase might be the result of the loss of E_2_ by ovariectomy and it suggests that E_2_ protects against the development of IR and hyperinsulinemia. E_2_ might increase the action of insulin in different organs and the lack of E_2_ might result in IR [31].

### 4.3. Systolic Blood Pressure

The changes observed in SBP could be related to several factors: (a) chronic ingestion of sucrose that causes an increase of 10–15 mmHg the SBP in rats [32], (b) IR and hypertriglyceridemia which are associated with impaired endothelial nitric oxide synthase (eNOS) activity and increased production of endothelin-1 [33], (c) hyperinsulinemia that increases circulating levels of free fatty acids which participate in the development of endothelial dysfunction [34], and (d) the loss of E_2_ by ovariectomy. Accordingly, the basic and clinical studies have reported elevations in SBP in postmenopausal women [35].

### 4.4. Renal Function

Endothelial cells are the main cell type found in the renal capillaries of the microcirculation and they participate in the conservation of renal hemodynamics and regulation of SBP [36]. MS might cause endothelial dysfunction in the renal microcirculation by increasing the Ca^2+^ influx and by elevating the number and sensitivity of receptors to vasoconstrictor substances in smooth muscle cells [37]. The endothelial dysfunction in the renal microcirculation has been associated with alterations in mesangial expansion which might contribute to the development of glomerulosclerosis, characterized by albuminuria, and a decline in creatinine clearance [38]. Our results suggest that there is deterioration of the renal function in MSovx rats which is evidenced by albuminuria and low creatinine clearance. This deterioration could be associated to the decrease in the E_2_ concentration [37]. Supplementation with E_2_ prevents albuminuria and improves the decline in glomerular filtration rate and renal function in MS [7]. Another study in which castrated female Wistar rats with 5/6 nephrectomy were studied showed mesangial expansion and a lower creatinine clearance [36]. In addition, E_2_ decreases the synthesis and the release of vasoconstrictor prostaglandins [4]. Our results show that the diminution of the E_2_ levels in the MSovx rats enhances vascular contraction in the isolated kidney. This effect is probably mediated by AA metabolites.

### 4.5. PLA2 Expression and AA of the Phospholipids

Diverse studies have shown that changes in the renal metabolism of AA may contribute to alterations in renal function in MS and hypertension [29, 37]. AA exists in the cell membrane phospholipids in esterified form [4]. Cellular levels of available AA for eicosanoids production are primarily controlled by PLA_2_ [13]. A proinflammatory state such as that present in renal damage may cause alterations in membrane lipid packaging and asymmetry which might increase PLA_2_ [39]. In this work we did not observe changes in the expression of PLA_2_ in any of our experimental groups; however, the percentage of AA of the phospholipids in the kidney homogenate was decreased in MSovx group. Although more research is required in this area of investigation, the activity of PLA_2_ could be increased. The reduction in the percentage of AA in the phospholipids could be related to an increase in the employment of PLA_2_ by the COX-2 and CYP450 4A enzymes, since they metabolize the eicosanoid vasoconstrictor substances. This would contribute to the observed increase of the SBP [4, 29]. Furthermore, no changes in PLA_2_ levels with treatment with E_2_ in ovx rats, sheep, and intact MS male rats have been found in other investigations [40, 41].

### 4.6. Δ Perfusion Pressure in Isolated Kidney and 5-LOX

There are very few reports on the role of 5-LOX in the regulation of the vascular tone and the participation of sex hormones in renal function. The administration of AA increased the ΔPP in the perfused isolated kidney of the MSovx group and the percentage of inhibition with Baicalein reached levels higher than 50%. These results suggest that E_2_ regulates renal vascular resistance and that the 5-LOX pathway may participate in the increase of the ΔPP observed in MSovx rats. The 5-LOX enzyme catalyzes oxygenation of AA to leukotrienes, HETEs, and lipoxins [42]. Glomerular mesangial cells, cortical tubules, and endothelial cells express LOX enzymes and the metabolites of this pathway regulate renal hemodynamics and glomerular filtration rates [29, 42]. They are also involved in the regulation of inflammation in the MS rats [38]. A study showed that LTs inhibited the production of PGI_2_ in the vascular endothelium and indirectly contributed to overall vascular constriction [42]. Another study in the perfused kidney of streptozotocin diabetic rats showed that Baicalein failed to modify renal vasoreactivity [43]. The authors concluded that LOX-derived metabolites played no role in the modulation by AA of the vascular response of the kidney [41]. However, another study in the rat kidney showed that 5-LOX was expressed 5 to 12 hours after injury by ischemia-reperfusion I/R [44]. Our results show that the expression of 5-LOX was diminished in kidney homogenate of MSovx group. However, the activity of this enzyme might not be impaired and it might participate in the regulation of renal vascular tone.

### 4.7. Δ Perfusion Pressure in Isolated Kidney and CYP450 4A

The CYP450 is expressed in the renal vasculature and in the nephron [45]. This enzyme metabolizes AA through three types of enzymatic reactions: arilic oxidation that gives rise to HETEs, epoxydation that metabolizes 5,6-, 8,9-, 11,12-, and 14,15-EETs, and spontaneous hydrolysis which produces DHTs and 19-, 20-HETEs [37, 45]. In spontaneously hypertensive rats (SHR), elevation of blood pressure in the nephron is associated with an increase in the expression of the CYP450 4A gene isoform [35]. In hypertension, androgen administration in ovx and intact female rats induces the expression of the CYP450 4A12 gene and it increases the biosynthesis of 20-HETE in proximal tubules [46]. This results in an elevation of albuminuria and the loss of creatinine clearance [45]. We found that the ΔPP was inhibited by Miconazole in more than a 90% in MSovx rats. The percentage of inhibition and overexpression of this enzyme could be related to the loss of E_2_, since this hormone downregulates the expression of the CYP450 4A2 and 4A8 genes. It thus causes a decrease in 20-HETE [38]. Elevation of CYP450 4A expression in preglomerular microvessels elevates renovascular resistance, reducing intravascular pressures in the medullary circulation thus promoting movement of fluid into the vasa rectae with expansion of extracellular fluid volume and elevation of SBP [36, 46, 47].

Our results suggest that, in the MSovx rats, the participation of the CYP450 4A pathway in AA metabolism is altered in a greater proportion than the 5-LOX pathway and that the presence of E_2_ is important in modulating these pathways in MS female rats. In this study we inhibited COX-2 with indomethacin, a nonspecific inhibitor, to assess the involvement of the 5-LOX and CYP450 4A pathways without its participation. An association between LOX and CYP450 metabolites and COX-2 pathway has been reported in MS and hypertension [48]. 20-HETE may be metabolized by COX-2 to PGH_2_ [38]. Our results show that the percentage of inhibition was greater than 100% in MS, MSovx, and MSovx + E_2_ groups. Previous investigations from our laboratory had shown that ovariectomy in MS female rats caused overexpression of renal COX-2. This was associated with a significant increase in PGE_2_ and TXB_2_ due to the decrease caused by the E_2_ treatment [4]. The loss of E_2_ might modulate the activity and the expression of the COX-2 pathway and the AA metabolism as postulated by other authors [49].

### 4.8. Cytokines

Cytokines are associated with MS and contribute to the progression of renal disease in this syndrome [50]. IL-1 and TNF-*α* are synthesized by macrophages that infiltrate organs with chronic inflammation [51]. IL-10 and IL-4 are anti-inflammatory cytokines that block the synthesis of IL-1 and TNF-*α*. E_2_ has potent anti-inflammatory properties and suppresses the expression of TNF-*α* and the increase of IL-10 [52]. Our results show that TNF-*α* was increased in the MS group in comparison to the C group and it was further increased in the MSovx group. The treatment with E_2_ in MSovx + E_2_ was not able to reverse this elevation. However, there was a decrease in IL-10 in the MSovx group in comparison to MS and MSovx + E_2_ groups. This suggests that the loss of E_2_ favors a proinflammatory state in the kidney of the MS rats. The overexpression of CYP450 4A has been associated with changes in TNF-*α* and IL-6. The CYP2J2 isoform that metabolizes vasodilator EETs also decreases TNF-*α* and induces the expression of cytokines and adhesion molecules in endothelial cell [52].

Finally, our histological studies made evident the structural changes present in MSovx. These effects are due to the development of MS while E_2_ seems to slow the development of the disease.

### 4.9. Limitations

The present investigation provides information on the effect of the loss of E_2_ by ovariectomy on the 5-LOX and CYP450 4A enzymes in kidney of MS female rats. Ovariectomy mimics the effect of menopause on the enzymatic pathways studied. During menopause, the production of sex hormones such as estrogens and progesterone decreases while the production of follicle-stimulating (FSH) and luteinizing hormones (LH) increases in an attempt to promote sex hormone production [53]. This increase in FSH and LH elevates the male hormone, testosterone [54]. These alterations in hormone production cause disorders in organs and systems that aggravate the possible pathologies present in the female sex.

## 5. Conclusions

In conclusion, there is a strong association between MS and renal dysfunction. This association is mediated by alterations in the AA metabolism. It is mediated through the 5-LOX and CYP450 4A pathways which are highly influenced by E_2_. The loss of E_2_ increases renal damage and treatment with this sex hormone benefits renal function by modulating AA metabolism through the 5-LOX and CYP450 4A pathways.

## Figures and Tables

**Table 1:** General characteristics in experimental groups.

Variables	C	MS	MSovx	MSovx + E_2_
SBP (mmHg)	117.1 ± 1.0	134.0 ± 0.5^**∗∗∗**^	141.2 ± 1.7^**∗∗∗**^	134.4 ± 0.8
Body weight (g)	239.0 ± 6.0	310.4 ± 12.0^**∗∗**^	445.0 ± 19.0^**∗∗∗**^	295.9 ± 4.4
Intra-abdominal fat (g)	4.8 ± 0.5	7.1 ± 0.4^**∗**^	13.3 ± 0.7^**∗∗∗**^	6.0 ± 0.1
Cholesterol (mg/dL)	37.5 ± 4.1	38.8 ± 2.1	39.4 ± 1.5	32.2 ± 5.8
Triglycerides (mg/dL)	105.9 ± 1.1	126.0 ± 3.3^**∗∗∗**^	147.3 ± 3.1^**∗∗∗**^	124.8 ± 4.2
Glucose (mmol/L)	4.4 ± 0.4	5.4 ± 0.3	6.6 ± 0.3	4.3 ± 0.3
Insulin (*μ*UI/L)	0.4 ± 0.1	5.1 ± 0.6^**∗∗∗**^	6.7 ± 0.4^**∗∗∗**^	4.9 ± 0.3
HOMA index	0.1 ± 0.0	1.6 ± 0.1^**∗∗∗**^	1.9 ± 0.1^**∗∗**^	0.8 ± 0.1
Estradiol (pg/mL)	24.8 ± 3.2	29.1 ± 5.8	9.2 ± 1.1^**∗∗∗**^	26.8 ± 3.6
Kidney weight (g)	1.0 ± 0.0	0.8 ± 0.0	0.8 ± 0.0	1.0 ± 0.1
Albuminuria (mg/24 hours)	3.4 ± 1.8	1.1 ± 2.4	15.7 ± 3.8^**∗**^	3.3 ± 4.8
Creatinine clearance (mL/min)	0.04 ± 0.003	0.03 ± 0.003	0.02 ± 0.002^**∗∗**^	0.03 ± 0.008

Data are means ± SE; *n* = 7 for each group. Statistically significant at C versus MS ^*∗*^ *p* < 0.05; ^*∗∗*^ *p* < 0.01; ^*∗∗∗*^ *p* < 0.001 and MS and MSovx + E_2_ versus MSovx ^*∗*^ *p* < 0.05; ^*∗∗*^ *p* < 0.01; ^*∗∗∗*^ *p* < 0.001. C: control; MS: metabolic syndrome; MSovx: metabolic syndrome ovariectomized; MSovx + E_2_: metabolic syndrome ovariectomized plus estradiol.

**Table 2:** Cytokines in the kidney homogenates of the experimental rat groups.

Cytokine (ng/100 *μ*g) protein	C	MS	MSovx	MSovx + E_2_
IL-1*α*/IL-1F1	0.02 ± 7 × 10^−4^	0.02 ± 1 × 10^−3^	0.03 ± 4 × 10^−3^	0.02 ± 3 × 10^−3^
IL-4	0.16 ± 1 × 10^−4^	0.14 ± 1 × 10^−4^	0.12 ± 5 × 10^−3^	0.14 ± 9 × 10^−3^
IL-10	0.63 ± 5 × 10^−3^	0.62 ± 6 × 10^−3^	** 0.03 ± **1 × 10^−3**∗∗**^	** 0.33 ± **1 × 10^−2**∗**^
TNF-*α*	0.23 ± 3 × 10^−3^	**0.74 ± **8 × 10^−3**∗∗**^	** 1.27 ± **5 × 10^−3**∗∗**^	** 1.23 ± **7 × 10^−3†^

Data are means ± SE; *n* = 7 for each group. Statistically significant at MSovx versus MSovx + E_2_^**∗**^ *p* < 0.01, ^**∗∗**^C and MSovx versus MS *p* < 0.001, and ^†^MS versus MSovx + E_2_ *p* < 0.001.
